# The content of soyasaponin and soyasapogenol in soy foods and their estimated intake in the Japanese

**DOI:** 10.1002/fsn3.107

**Published:** 2014-03-30

**Authors:** Shuichi Kamo, Shunsuke Suzuki, Toshiro Sato

**Affiliations:** Fine Chemical Laboratory, J-OIL MILLS, Inc.1746, Nakashinden, Fukuroi-shi, Shizuoka, 437-1111, Japan

**Keywords:** HPLC–MS/MS, soy foods, soyasapogenols, soyasaponins

## Abstract

Soyasaponins have been reported to promote various health functions. However, the total soyasaponin and soyasapogenol content in soy products and the daily intake remain to be fully elucidated. We developed a high-performance liquid chromatography coupled with tandem mass spectrometric (HPLC–MS/MS) method to evaluate the content of group A and B soyasaponins and soyasapogenols. The total soyasaponin content was measured after pretreatment converted soyasaponins to soyasapogenols. The total soyasaponin content in soy foods was 200–1800 nmol g^−1^, although that of soy sauce was 2–7 nmol g^−1^. The soyasapogenol to total soyasaponin ratio was 30–50% in long-term matured miso. The majority of the soyasapogenol detected was soyasapogenol B rather than soyasapogenol A, resulting in speculation that further steps are required to liberate aglycones from glycoside-conjugated soyasaponins in soyasapogenol A. We estimated the daily intake of total soyasaponins and soyasapogenols by the Japanese, which was 50.3 and 0.59 *μ*mol, respectively. The soyasapogenol content and the soyasapogenol to total soyasaponin ratio was considerably low in most soy products, except for long-term maturated miso. The major source of the daily intake of soyasaponins and soyasapogenols were tofu and miso, respectively.

## Introduction

Soyasaponins have been reported to promote various health functions because of their antioxidative (Ishii and Tanizawa 2006) and cholesterol-lowering properties (Lee et al. 2005), reduced blood glucose levels (Tanaka et al. 2006), anti-kidney disease progression (Philbrick et al. 2003) and anti-inflammatory properties (Lee et al. 2010), renin inhibition (Takahashi et al. 2008), hepatoprotection (Kinjo et al. 1998), and antitumor effects (Kerwin 2004; Ellington et al. 2005; Zhang and Popovich 2010).

**Figure 1:**
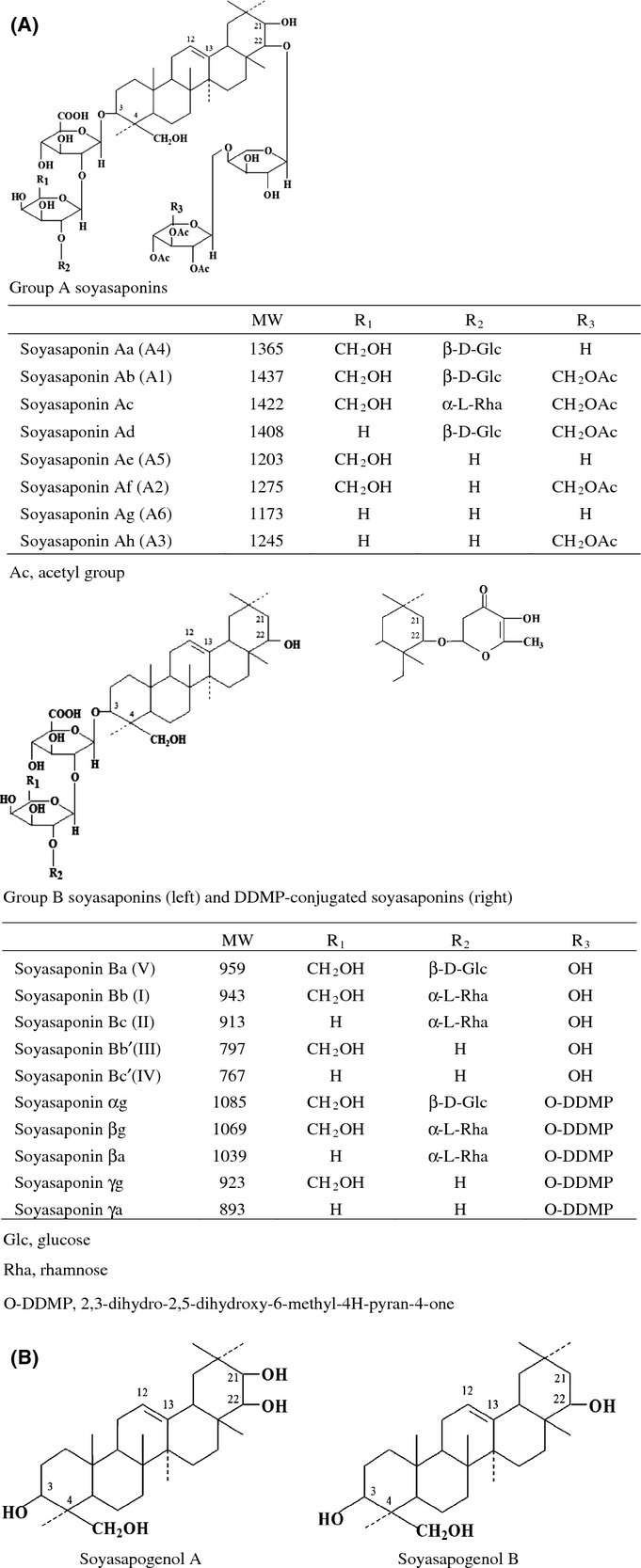
Chemical structures of group A soyasaponins, group B soyasaponins, soyasapogenol A, and soyasapogenol B. (A) Chemical structure of group A soyasaponins and group B soyasaponins. (B) Chemical structures of soyasapogenol A and soyasapogenol B.

Soyasaponins are triterpenoid glycosides that possess an oleanane-type aglycone with polysaccharide chains. Soyasaponins are mainly classified into group A and group B soyasaponins. Group A soyasaponins are glycosylated at the C-3 and C-22 position of soyasapogenol A, whereas group B soyasaponins, including 2,3-dihydro-2,5-dihydroxy-6-methyl-4H-pyran-4-one (DDMP), are glycosylated at the C-3 position of soyasapogenol B (Fig. 1). Each group consists of many homologs. Group A soyasaponins include eight compounds that differ with respect to the number of glycosides attached to soyasapogenol A (Fig. 1). Various isomers of group A soyasaponins also exist, which differ in the number of acetyl groups attached to the glycosides in each group A soyasaponin compound.

Several methods have been reported for quantifying soyasaponins in soy. Jin et al. (2006) developed a high-performance liquid chromatography (HPLC) method coupled with electrospray mass spectrometry (MS) to quantify over 50 homologs of soyasaponins. However, corresponding soyasaponin standards were not used when the soyasaponin content was measured according to the method of Jin et al. (2006). Murphy et al. (2008) reported the group B soyasaponin content in soy foods and soy ingredients; including soy milk, soy infant formula, tofu, and fermented soy foods such as miso, tempeh, and natto; and found that most soy foods contained about >1 *μ*mol g^−1^ of group B soyasaponin, with some exceptions. However, they did not measure the group A soyasaponin content, which is also a major soyasaponin compound. Furthermore, the content of aglycone forms, soyasapogenols, which are expected to be formed in fermented soy foods, was also not measured. Hu et al. (2004) reported that soyasaponin I, a group B soyasaponin compound, was degraded into soyasapogenol B in the presence of human fecal microflora. Recently, we found that soyasapogenol is absorbed into blood after the conversion of soyasaponin into soyasapogenol in rats (Kamo et al. 2014). In addition, we reported that soyasapogenols are much better absorbed than corresponding soyasaponins, and group B soyasaponin is better absorbed than group A soyasaponin (Kamo et al. 2014). Thus, soyasapogenols are expected to have more potent health-promoting effect, but the total soyasaponin and soyasapogenol content in soy foods remain to be fully elucidated.

The Japanese consume many soy products that are believed to confer health benefits. Determining the intake of soyasaponins is important for evaluating their health benefits and safety. However, the Japanese intake levels of soyasaponins and its aglycones, soyasapogenols, have not been clarified. Thus, we developed a method for measuring the content of group A soyasaponins, group B soyasaponins, soyasapogenols, and total soyasaponin in soy foods using HPLC–MS/MS. We also estimated the daily intake of total soyasaponins and soyasapogenols on the basis of the soyasaponin content in soy foods and a survey of the daily intake of soy foods by the Japanese (Ministry of Health, Labour and Welfare 2005).

## Materials and Methods

### Reagents

Soyasapogenol A and B (purity > 98%) were purchased from Tokiwa Phytochemical (Chiba, Japan). Solvents and other chemicals were either HPLC grade or high quality.

### Soy foods

Major brands of Japanese soy food products were purchased from local supermarkets and online stores. The number of analytical samples in each soy food product category was six for soy sauce, nine for miso (shiro = 4, aka = 5), five for natto, three for tofu, five for deep-fried tofu, four for toasted soybean flour, four for soymilk, and three for tempeh.

### Extraction from natto

A 20-g sample of natto was ground using a blender (Oster Co., Milwaukee, WI). The ground sample was sonicated in 50 mL of 70% (v/v) ethanol for 1 h. The supernatant was collected after centrifugation at 1500*g* for 15 min. This supernatant collection procedure was repeated twice. The supernatants were combined and concentrated using a rotary evaporator with reduced pressure. Next, 100 mL of water and 200 mL of ethyl acetate were added to the concentrated supernatant, the mixture was shaken in a separating funnel, and the upper layer (ethyl acetate layer) was retained. We added 200 mL of ethyl acetate to the residue, and the same procedure was repeated twice. The separated upper ethyl acetate fractions were combined and concentrated to 10 mL using a rotary evaporator with reduced pressure.

### Extraction from soy sauce

We added 400 mL of ethyl acetate to a 200-mL soy sauce sample, and the mixture was shaken in a separating funnel. The upper ethyl acetate fraction was then collected. Next, 200 mL of ethyl acetate was added to the residue, and the same procedure was repeated twice. The upper ethyl acetate fractions were combined and concentrated to 10 mL using a rotary evaporator with reduced pressure.

### Extraction from other soy foods

We ground 15–20-g samples of tofu, deep-fried tofu, and tempeh in 70% (v/v) ethanol using a blender. Next, we added 70% (v/v) ethanol to 3–5-g samples of miso, toasted soybean flour, and soy milk without grinding in a blender. Each sample was sonicated for 1 h, and the supernatant was collected after centrifugation at 1500*g* for 15 min. For tofu, deep-fried tofu, and tempeh, 50 mL of 70% (v/v) ethanol was added to the residue. For miso, toasted soybean flour, and soy milk, 100 mL of 70% (v/v) ethanol was added to the residue. The slurry mixture was sonicated, and the supernatant of the extract was collected after centrifugation again at 1500*g* for 15 min. The combined supernatant was concentrated to 50 mL using a rotary evaporator with reduced pressure.

### Analysis of the content of soyasapogenols and total soyasaponins and calculation of the proportion of soyasapogenols

After filtering the prepared sample, the content of soyasapogenol A and soyasapogenol B in soy foods was measured by HPLC–MS/MS.

The total soyasaponin content was calculated as follows. We added 10 mL of 10% (w/w) HCl–methanol to the concentrated sample and heated it at 80°C with refluxing for 2 h. The complete hydrolysis of soyasaponin was performed, and degraded soyasapogenol was hardly detected in this hydrolysis condition. The treated sample was diluted to 100 mL with methanol, and we measured the content of soyasapogenol A and soyasapogenol B by HPLC–MS/MS. The total soyasaponin content was determined on a mol basis (nmol g^−1^ wet weight) of soyasapogenol A and soyasapogenol B.

The ratio of original soyasapogenols in soy foods was calculated by dividing the original content of soyasapogenol A and soyasapogenol B in soy foods by the content of total soyasapogenol A and soyasapogenol B after treatment with HCl–methanol.

Soy food samples were analyzed for soyasaponins and soyasapogenols in duplicate. The average data of soy food samples in each group are presented as mean ± standard deviation (SD).

### HPLC–MS/MS analysis

The soyasaponin content of soy foods was determined as follows. The prepared methanol solution containing the evaporated extracts was filtered, and the filtrate was subjected to HPLC–MS/MS (ACQUITY UPLC and Quattro micro API; Waters, Milford, MA). HPLC analyses were conducted with a Waters ACQUITY UPLC system consisting of Binary Solvent Manager (pump and degasser), Sample Manager (auto sampler and column oven), and photo diode array (PDA) detector.

The mobile phases were (A) 0.05% formic acid in methanol and (B) 0.05% formic acid in water where (A):(B) = 90:10 operated at a flow rate of 0.2 mL min^−1^ in the isocratic mode. The separation was performed using a BEH shield RP18 column (2.1 mm × 150 mm, 1.7 *μ*m). The column temperature was 40°C. The total sample run time was 5 min. The injection volume was 5 *μ*L.

Detection was operated using an electrospray ionized MS in the positive ion mode with the multiple reaction monitoring (MRM) mode. The source temperature and the desolvation temperature were 120°C and 350°C, respectively. The capillary voltage was 3 kV. The flow rate of N_2_ gas was 600 L/h. Helium gas was introduced into the trap to induce collisions with the precursor ion. An outline of the MRM parameters for soyasapogenols is provided in Table 1.

**Table 1:** Multiple reaction monitoring (MRM) parameters of soyasaponins and soyasapogenols

	Soyasapogenol A	Soyasapogenol B
Precursor ion (m/z)	457	441
Daughter ion (m/z)	95	95
Cone voltage (V)	28	28
Collision voltage (V)	23	28
Retention time (min)	3.6	4.1

The coefficient variation (CV) for soyasapogenol A (14.7 *μ*g mL^−1^) and B (17.2 *μ*g mL^−1^) standard solution peak area was 1.0% and 1.5%, respectively (*n* = 6).

### Calculation of daily intake of soyasaponin and soyasapogenol

We calculated the daily intake of total soyasaponin and soyasapogenol on the basis of results of the soyasaponin content analysis of soy foods and a survey of the daily soy foods consumption by the Japanese (http://www.mhlw.go.jp/bunya/kenkou/eiyou07/01.html).

To determine the approximate intake on a per weight basis, we calculated the molar levels of total soyasaponins. As soyasaponin A1 (MW = 1437) and soyasaponin I (MW = 943) are reported to be the major constituents of group A and group B soyasaponins, respectively (Goda et al. 2002; Tani et al. 1985), soyasaponin A1 and soyasaponin I were employed as the representatives of group A and group B soyasaponins, respectively.

## Results and Discussion

### Total soyasaponin and soyasapogenol content of soy foods

The existence of numerous homologs of soyasaponins in soy foods makes it difficult to analyze each compound until the current study; therefore, measurement of the total soyasaponin content has not been achieved. Thus, we attempted to determine the total soyasaponin content on a molar basis by converting each soyasaponin compound into soyasapogenols, which were analyzed using a highly sensitive HPLC–MS/MS method. We also measured the original content of the aglycone forms, soyasapogenol A and soyasapogenol B, in soy foods without pretreatment by hydrolysis. The total soyasaponin and soyasapogenol content of soy foods are shown in Table 2. The molecular weight of each soyasaponin compound was different; therefore, the soyasaponin content of soy foods were expressed as nmol g^−1^ to make the results comparable.

**Table 2:** Content of soyasaponin and soyasapogenol in soy foods and the ratio of soyasapogenol

		Soyasaponin content (nmol g^−1^ wet weight)			Soyasapogenol content (nmol g^−1^ wet weight)			Ratio of soyasapogenol^1^ (%)		
				
Moisture (%)		A	B	Total	A	B	Total	A	B	Total
Miso^2^										
A	41.8	68.2	370.0	438.2	–	2.6	2.6	–	0.7	0.6
B	33.7	46.0	198.8	244.8	–	1.3	1.3	–	0.7	0.5
C	32.6	256.8	542.7	799.5	–	1.5	1.5	–	0.3	0.5
D	39.4	360.4	695.0	1055.4	–	5.3	5.3	–	0.8	0.5
E	40.8	70.4	329.9	400.3	–	107.0	107.0	–	32.4	26.7
F	40.5	63.1	247.5	310.6	–	81.3	81.3	–	32.8	26.2
G	31.5	54.4	211.6	266.0	–	119.2	119.2	–	56.3	44.8
H	32.1	57.5	230.1	287.6	–	75.4	75.4	–	32.8	26.2
I	36.3	97.3	346.4	443.7	–	17.2	17.2	–	5.0	3.9
(Mean ± SD)		119.3 ± 111.3	352.4 ± 167.3	471.8 ± 275.9	–	45.6 ± 49.4	45.6 ± 49.4	–	18.0 ± 20.9	14.4 ± 16.7
Natto										
A	59.3	226.1	774.7	1000.8	–	0.02	0.02	–	0.003	0.002
B	58.8	163.3	818.3	981.6	–	0.01	0.01	–	0.001	0.001
C	58.1	264.4	795.6	1060.0	–	–	–	–	–	–
D	58.9	228.6	738.7	967.3	–	–	–	–	–	–
E	58.6	239.8	774.9	1014.7	–	0.07	0.07	–	0.008	0.006
(Mean ± SD)		224.4 ± 37.4	780.4 ± 29.4	1004.9 ± 35.7	–	0.03 ± 0.03	0.03 ± 0.03	–	0.004 ± 0.004	0.003 ± 0.003
Tofu^3^										
A	84.8	120.7	547.5	668.2	–	–	–	–	–	–
B	84.7	220.4	430.4	650.8	–	–	–	–	–	–
C	82.3	143.5	369.3	512.8	–	–	–	–	–	–
(Mean ± SD)		161.5 ± 52.2	449.1 ± 90.6	610.6 ± 85.1	–	–	–	–	–	–
Deep-fried tofu										
A	52.1	262.6	1168.0	1430.6	–	1.13	1.13	–	0.10	0.08
B	44.3	614.9	952.3	1567.2	–	0.61	0.61	–	0.06	0.04
C	42.3	358.7	1227.9	1586.6	–	0.65	0.65	–	0.05	0.04
D	37.9	504.7	1326.5	1831.2	–	0.78	0.78	–	0.06	0.04
E	60.8	424.2	1186.3	1610.5	–	0.24	0.24	–	0.02	0.01
(Mean ± SD)		433.0 ± 135.0	1168.7 ± 137.4	1605.2 ± 144.4	–	0.68 ± 0.32	0.68 ± 0.32	–	0.06 ± 0.03	0.04 ± 0.02
Toasted soybean flour										
A		853.9	1781.0	2634.9	–	8.93	8.93	–	0.50	0.34
B		654.9	1477.3	2132.3	–	14.16	14.16	–	0.96	0.66
C		660.0	2376.9	3036.9	–	4.79	4.79	–	0.20	0.16
D		950.3	2705.2	3655.5	–	1.74	1.74	–	0.06	0.05
E		789.5	2467.8	3257.2	–	2.40	2.40	–	0.10	0.07
(Mean ± SD)		781.7 ± 127.1	2085.1 ± 512.0	2943.4 ± 584.7	–	6.41 ± 5.17	6.41 ± 5.17	–	0.36 ± 0.37	0.26 ± 0.25
Soy milk										
A	88.3	84.5	317.7	402.2	–	1.79	1.79	–	0.56	0.45
B	88.9	133.1	321.5	454.6	–	1.36	1.36	–	0.42	0.30
C	90.0	76.8	246.2	323.0	–	0.47	0.47	–	0.19	0.15
(Mean ± SD)		98.1 ± 30.5	295.1 ± 42.4	393.3 ± 66.3	–	1.21 ± 0.67	1.21 ± 0.67	–	0.39 ± 0.19	0.30 ± 0.15
Tempeh										
A	63.2	185.0	461.1	646.1	–	1.58	1.58	–	0.34	0.24
B	47.9	267.2	701.0	968.2	–	–	–	—	–	–
C	55.6	942.2	593.0	1535.2	–	0.98	0.98	–	0.17	0.06
D	55.6	150.5	859.9	1010.4	0.76	2.66	3.42	0.50	0.31	0.34
E	55.6	183.6	261.6	445.2	–	1.15	1.15	–	0.44	0.26
(Mean ± SD)		345.7 ± 336.2	575.3 ± 228.3	921.0 ± 415.2	–	1.59 ± 0.76	1.78 ± 1.12	–	0.31 ± 0.11	0.23 ± 0.12
Soy sauce^4^										
A		1.68	0.65	2.33	0.006	0.048	0.054	–	7.4	2.3
B		2.53	1.28	3.81	–	0.009	0.009	–	0.7	0.2
C		1.87	0.33	2.20	–	0.017	0.017	–	5.2	0.8
D		4.50	0.78	5.28	–	0.009	0.009	–	1.2	0.2
E		4.45	1.35	5.80	–	0.009	0.009	–	0.7	0.2
F		2.40	4.65	7.05	0.002	0.037	0.039	–	0.8	0.6
(Mean ± SD)		2.91 ± 1.26	1.51 ± 1.59	4.41 ± 1.96	–	0.022 ± 0.017	0.023 ± 0.019	–	2.6 ± 2.9	0.7 ± 0.8

1 The ratio of soyasapogenol was calculated based on the mol of soyasapogenol/the mol of soyasaponin. – = not detected.

2 Miso: A–D = shiro (white), E–I = aka (red).

3 Tofu = A, C (momen), B (kinugoshi).

4 Soy sauce = A and B, C and D, E and F, where the pairs are from the same manufacturers. A, C, and E (ingredient: defatted soy bean), B, D, F (ingredient: nondefatted soy bean).

### Conversion of soyasaponins into soyasapogenols in miso

Miso is a paste-type seasoning that is fermented by *Lactobacillus* and *Aspergillus oryzae*. Shiro (white) and aka (red) are well-known types of miso. Shiro is characterized by its white color, and it has a short-term fermentation (normally 1 month) during maturation. In contrast to shiro, aka is characterized by its dark color and it has a long-term fermentation (normally more than 3 months) during maturation. The average content of the total soyasaponins in miso was 471.8 nmol g^−1^, which had little relation with the manufacturing process of miso. Soyasapogenol B was detected in both types of miso, but the ratio of soyasapogenol B to group B soyasaponin was higher in aka miso. The long-term fermentation and maturation process with aka miso appeared to promote the conversion of group B soyasaponins into soyasapogenol B. However, soyasapogenol A was not detected in miso. The carbohydrate structure of group A soyasaponins is more complex than that of group B soyasaponins, so the full removal of the carbohydrate moiety appeared to hardly occur during the manufacturing process. Murphy et al. (1999) reported that the ratios of soy isoflavone aglycones in shiro and aka were 29.0% and 39.4%, respectively. The glycoside moiety of soy isoflavone is simple and it appeared to be easily removed during the short-term fermentation and maturation process with shiro miso.

### Conversion of soyasaponins into soyasapogenols in natto and tempeh

Natto is a soy food, which is fermented by *Bacillus subtilis* natto. The average content of total soyasaponins in natto was 1004.9 nmol g^−1^. Tempeh is a traditional Indonesian soy food, which is fermented by *Rhizopus*. The average content of total soyasaponins in tempeh was 921.0 nmol g^−1^. Only a small amount of soyasapogenol B was detected in these fermented products, which suggests that the types of microorganism and the time taken for fermentation affected the conversion of soyasaponins into soyasapogenols.

### Conversion of soyasaponins into soyasapogenols in soy sauce

Soy sauce is a liquid seasoning, which is fermented by *Lactobacillus*, *Saccharomyces cerevisiae*, *A. oryzae*, and other organisms. The average content of total soyasaponin in soy sauce was 4.4 nmol g^−1^. Soyasapogenol B and soyasapogenol A were both detected in soy sauce. With soy sauce being a liquid product, most of the hydrophobic compounds such as soyasapogenols may be removed during the manufacturing process.

### Conversion of soyasaponins into soyasapogenols in other soy foods

The average content of total soyasaponins in tofu, deep-fried tofu, toasted soybean flour, and soy milk were 1004.9, 1605.2, 2943.4, and 393.3 nmol g^−1^, respectively. The manufacturing processes of deep-fried tofu, toasted soybean flour, and soy milk do not involve a fermentation process, but small amounts of soyasapogenol B were detected, which suggests that the hydrolysis of the polysaccharide chain in group B soyasaponins occurred during the heat treatment process.

### Comparison of soyasaponin contents in previous reports

Murphy et al. (2008) reported that the average group B soyasaponin content of seven types of tofu was 4.4 *μ*mol g^−1^, on a dry mass basis. Hu et al. (2004) reported that the average group B soyasaponin content of tofu and soymilk were 4.5 and 5.1 *μ*mol g^−1^ (on a dry mass basis), respectively. Kitagawa et al. (1984) reported that average group B soyasaponin content of tofu and natto were approximately 2.03–2.09 *μ*mol g^−1^ and 1.78–1.84 *μ*mol g^−1^, respectively. Ireland et al. (1986) reported that average total soyasaponin content of soy milk was 4.0 *μ*mol g^−1^. Thus, our results were similar to the ranges in these previous reports.

### Calculation of the daily intake of total soyasaponin and soyasapogenol in the Japanese

**Figure 2:**
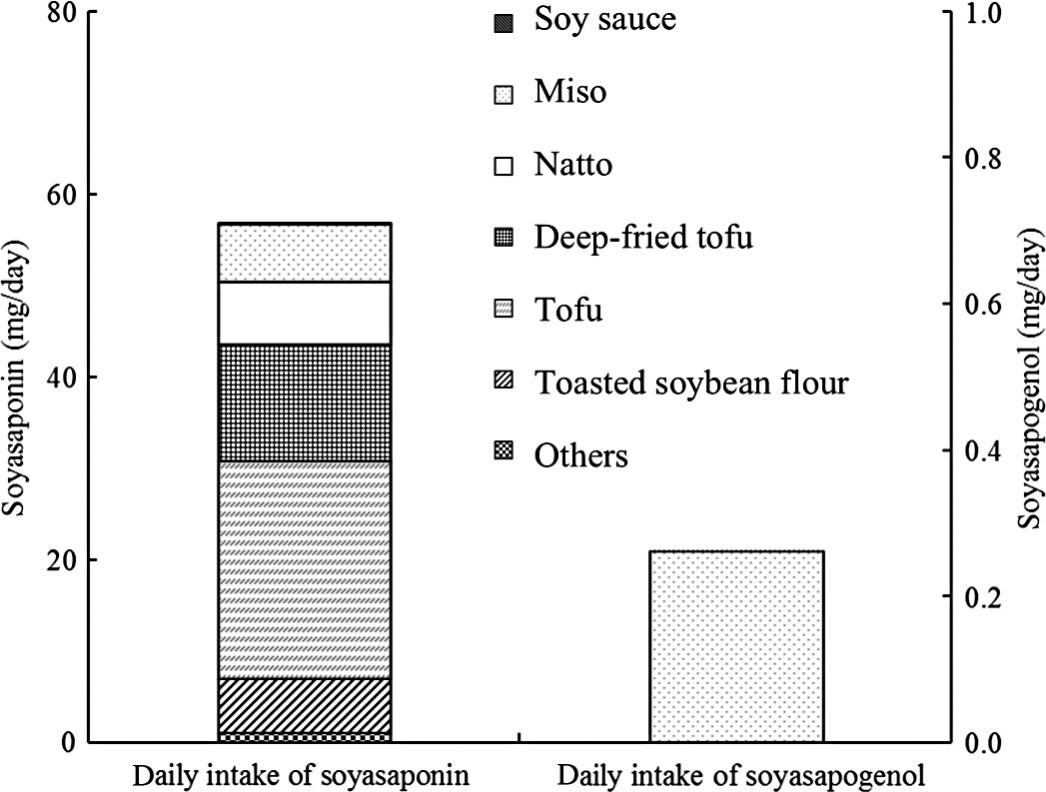
Average daily intake of soyasaponins and soyasapogenols in the Japanese. The daily intake of soyasaponins (left axis) and the daily intake of soyasapogenols (right axis). The daily intake of soy foods is derived from The National Health and Nutrition Survey, Ministry of Health, Labour and Welfare (2005)

The daily average soy foods consumption levels in the Japanese were as follows: tofu = 36.3 g, natto = 6.5 g, miso = 12.5 g, deep-fried tofu = 7.4 g, soy sauce = 17.4 g, toasted soybean flour = 1.9 g, and others (including soy milk and tempeh) = 1.4 g. As shown in Figure 2, the intakes of group A soyasaponins, group B soyasaponins, and total soyasaponins were estimated to be 13.9 *μ*mol day^−1^ (20.0 mg day^−1^ soyasaponin A1 equivalent), 39.1 *μ*mol day^−1^ (36.8 mg day^−1^ soyasaponin I equivalent), and 53.0 *μ*mol day^−1^ (56.8 mg day^−1^ soyasaponin A1 and I equivalent combined), respectively. The types and amounts of soy foods consumed varied among individuals. Several surveys of soy isoflavone intake from soy foods have been conducted. The average intake of soy isoflavones was found to be 39.46 mg (7.8–87.7 mg) (Kimira et al. 1998). Japanese government research has shown that the variation in soy foods intake is quite high. The estimated intake value for the 95th percentile was 2.8 times higher than the average intake (Ishimi 2001). The variation in the soyasaponin intake appeared to be at the same degree to that of soy isoflavone.

The average intake of soyasapogenol was 0.59 *μ*mol day^−1^ (271 *μ*g day^−1^), most of which was in the form of soyasapogenol B. Only 11.0% of the source of soyasaponin was accounted for in miso, whereas most of the soyasapogenol (96.6%) was accounted for in miso. The type of miso preferred varied among the areas in Japan and the daily intake of soyasapogenol B in aka miso-consuming areas was estimated to be higher.

Soyasaponins have been reported to promote various health functions, but the effects of soyasaponin intake have been unclear until the present study. Recently, we found that group B soyasaponin is better absorbed than group A soyasaponin, and soyasapogenol B is further better absorbed than group B soyasaponin (Kamo et al. 2014). The required dose for each health function and the bioavailability of each type of soyasaponin homolog should be clarified in further studies.

## Conclusions

We developed a high-performance liquid chromatography coupled with tandem mass spectrometric (HPLC–MS/MS) method for measuring soyasaponins and soyasaponin aglycones (soyasapogenols) in soy products. The total soyasaponin content of soy foods ranged from 244.8 to 2943.4 nmol g^−1^ although that of soy sauce ranged from 2.20 to 7.05 nmol g^−1^. The total soyasapogenol content ranged from 0.009 to 119.2 nmol g^−1^. The soyasapogenol to total soyasaponin ratio was quite low in most of soy foods except long-term maturated miso. Majority of the soyasaponin aglycone detected in soy foods was soyasapogenol B rather than soyasapogenol A, speculating that further steps were required to convert glycoside-conjugated soyasaponins into soyasaponin aglycones in soyasapogenol A. The average daily intake of total soyasaponins and soyasapogenols was estimated to be 53.0 *μ*mol and 0.59 *μ*mol, respectively.
